# Expression of concern: ‘RNA editing of microRNA prevents RNA-induced silencing complex recognition of target mRNA’ (2015), by Cui Y *et al.*

**DOI:** 10.1098/rsob.230300

**Published:** 2023-08-31

**Authors:** Open Biology editorial team

Following the publication of ‘RNA editing of microRNA prevents RNA-induced silencing complex recognition of target mRNA’, the Editorial team have concerns regarding the validity of the data used in this study.

We are issuing an expression of concern here and investigating this as a matter of urgency; we will notify readers as to the results of our investigation as soon as possible.

